# Fine Mapping and Candidate Gene Identification for the *CapUp* Locus Controlling Fruit Orientation in Pepper (*Capsicum* spp.)

**DOI:** 10.3389/fpls.2021.675474

**Published:** 2021-06-28

**Authors:** Abate Mekonnen Solomon, Tae-Gun Kim, Koeun Han, Hea-Young Lee, Abhinandan Patil, Muhammad Irfan Siddique, Jeonghwan Ahn, Byoung-Cheorl Kang

**Affiliations:** ^1^Department of Agriculture, Forestry and Bioresources, Research Institute of Agriculture and Life Sciences, Plant Genomics Breeding Institute, College of Agriculture and Life Sciences, Seoul National University, Seoul, South Korea; ^2^Interdisciplinary Program in Agricultural Genomics, Department of Agriculture, Forestry and Bioresources, Research Institute of Agriculture and Life Sciences, Plant Genomics Breeding Institute, College of Agriculture and Life Sciences, Seoul National University, Seoul, South Korea; ^3^ECOSEED, Gimje-si, South Korea

**Keywords:** pepper, fruit orientation, fine mapping, candidate gene, *up* gene

## Abstract

The orientation of fruits is a distinguishing morphological feature of pepper (*Capsicum* spp.) varieties. The pendent (downward curved) growth of the fruit stalks, known as pedicels, is highly correlated with fruit weight and pedicel length. A previous genetic analysis revealed that the pendent fruit orientation is governed by a dominant gene, and incomplete inheritance is also observed in some *Capsicum* accessions. To identify and localize this gene, a single quantitative trait locus (QTL) analysis was performed on one F_2_ and two recombinant inbred line (RIL) populations, and a genome-wide association study (GWAS) was performed using a core collection. Common QTL regions associated with fruit orientation were detected on chromosome 12. A total of 187,966 SNPs were identified in a genotyping-by-sequencing (GBS) for GWAS analysis of 196 *Capsicum annuum*, 25 *Capsicum baccatum*, 21 *Capsicum chinense*, and 14 *Capsicum frutescens* accessions, representing the germplasm collection of South Korea. The results of these analyses enabled us to narrow down the *CapUp* region of interest to 200–250 Mbp on chromosome 12. Seven candidate genes were found to be located between two markers that were completely cosegregated with the fruit orientation phenotype. The findings and markers developed in this study will be helpful for additional understanding of pepper fruit development and breeding for fruit orientation.

## Introduction

Peppers (*Capsicum* spp.) originated in the New World of Central and South America, and are now one of the most important cultivated crops in the Solanaceae family (Bai and Lindhout, 2007; Li et al., 2013). Five pepper species, *Capsicum annuum*, *Capsicum frutescens*, *Capsicum baccatum*, *Capsicum chinense*, and *Capsicum pubescens*, were domesticated over 6,000 years ago (Bai and Lindhout, 2007; Li et al., 2013). Initially, peppers were used as food preservatives and medicines, but were subsequently consumed as a spice and a vegetable in their own right (Chunthawodtiporn et al., 2018). The wild forms of pepper are small and produce soft, red, pungent, and deciduous (easy to separate from the calyx) fruits, in addition to their small and pubescent leaves; however, selection during domestication resulted in non-deciduous cultivars with large and less pungent fruits of different colors and hairless leaves.

The change in fruit position from erect, in which the fruit is held in an upright position, to pendent, where the fruits are pendulous or hang freely, has also been described as an important step in pepper selection and domestication (Paran and Van Der Knaap, 2007; Albrecht et al., 2012; Chunthawodtiporn et al., 2018). This change in fruit orientation may be associated with an increase in fruit size and length, and/or a decrease in the thickness of the pedicel (a short stem-like organ that links the flower/fruit to the inflorescence axis), as well as better protection from sun exposure and predation by birds (Setiamihardja and Knavei, 1990; Paran and Van Der Knaap, 2007). There is a specific market in some parts of pepper growing regions where the erect fruit type is more important than the pendent type. Since the erect phenotype is controlled by a recessive gene, development of molecular markers is necessary for breeding erect type cultivars.

The straight or curved growth of the pedicel governs the orientation of fruits to become either erect or pendent, respectively. This phenomenon is known to be regulated by a controlled sequence of cell proliferation, differentiation, and elongation (Bundy et al., 2012). A study of pedicel growth in *Arabidopsis* has provided clues about the mechanisms by which the proximal constriction of the pedicel along the abaxial and lateral sides cause the downward bending of the distal pedicel (Douglas and Riggs, 2005). In capsule- and follicle-bearing plants, an erect fruit phenotype is required for the dispersal of seeds (Niu et al., 2016). In *Arabidopsis thaliana*, the *BREVIPEDICELLUS* (*BP*) gene regulates the proliferation of cells during pedicel elongation and influences the curvature of the abaxial region (Wang et al., 2015). The tobacco (*Nicotiana tabacum*) MADS-box gene *SHORT VEGETATIVE PHASE* (*NtSVP*) plays a role in the elongation and orientation of the pedicel (Wang et al., 2015). Similarly, the tomato (*Solanum lycopersicum*) gene *ARGONAUTE7* (*S1AGO7*) is responsible for the upward-pointing growth of the pedicels (Lin et al., 2016). Douglas et al. (2002) reported for Arabidopsis (*A. thaliana*) that *KNAT1* and *ERECTA* play a role in pedicel bending at the nodes and the resulting downward orientation of the flowers, proposing that this is caused by the loss of chlorenchyma tissue at the node adjacent to the lateral organs and in the abaxial regions of the pedicels. Later, the function of *LEAFY* (*LFY*) in the pendent growth of *Arabidopsis* pedicels was described; it functions alongside *BP* to reduce the cortical cell length in the abaxial domain (Yamaguchi et al., 2012). Consistent with these, *KNAT6* and *KNAT2* were shown to play a role in the downward-pointing phenotype of *Arabidopsis* inflorescences (Ragni et al., 2008).

Over the years, genetic studies have revealed that fruit orientation in pepper (*Capsicum annuum*) is a qualitative trait controlled by a single gene located on chromosome 12 (Kaiser, 1935; Cheng et al., 2016). An early study by Lee et al. (2008) used the Saengryeog 211 (pendent) and Saengryeog 213 (erect) cultivars, alongside their F_1_ and BC_1_ progeny, to demonstrate that the gene responsible for erect fruit, *up*, is recessive. These authors developed a fruit orientation–associated cleaved amplified polymorphic sequence (CAPS) marker, which was mapped at a genetic distance of 4.3 cM from the locus. In 2016, an ultra-high density bin mapping using a recombinant inbred line (RIL) population, derived from *C. annuum* ‘Perennial’ (erect) and ‘Dempsey’ (pendant), detected a major quantitative trait locus (QTL) associated with fruit orientation, *FP-12.2.* This QTL, residing at 199.6 Mbp on chromosome 12 in the CM334 reference genome, explained >40% of the phenotypic variation between genotypes (Han et al., 2016). However, the dissection and identification of the causal genes underlying this QTL is difficult due to the large size of this region.

In this study, we used two RIL and three F_2_ populations for a linkage analysis, combining the results with a genome-wide association study (GWAS) using a core collection, with the aims of reevaluating the genetic effect of the *CapUp* gene in a new population, fine mapping the *CapUp* locus. Through gene expression analysis and identification of gene variation, we inferred candidate genes for *CapUp*.

## Materials and Methods

### Plant Materials

This study included four *Capsicum annuum* F_2_ populations, which originated from crosses between: the erect ‘MicroPep’ and pendent ‘Jeju’ lines (219 F_2_ plants), the erect ‘Lp97’ and pendent ‘A79’ lines (379 F_2_ plants), and the erect ‘U92’ and pendent ‘A106’ lines (63 F_2_ plants), and erect ‘UB7’ and pendent ‘GB57’ lines (98 F_2_ plants). Two *C. annuum* RIL populations were also used, which were generated from the erect ‘Perennial’ and pendent ‘Dempsey’ lines (77 RILs) and the erect ‘35001 (F)’ and pendent ‘35009 (C)’ lines (174 RILs) obtained from Rural Development Administration (Wanju, South Korea). They are respectively coded hereafter as MJ, LA, UA, UG, PD, and FC, respectively, from the initials of their respective parents’ names. The parental lines Lp79, A79, U92, A106, UB7, and GB57 were provided by EcoSeed P.L.C., Gimje-si, Republic of Korea. MJ and two RIL populations were developed by our lab. A core collection composed of 196 *C. annuum*, 25 *C. baccatum*, 21 *C. chinense*, and 14 *C. frutescens* genotypes was used for the GWAS (Table 1; Lee et al., 2016).

**TABLE 1:** Segregation of fruit orientation in different pepper species in the core collection used for GWAS.

Species	Fruit orientation		Total
	Erect	Pendent	
*C. annuum*	57	139	196
*C. baccatum*	4	21	25
*C. chinense*	1	20	21
*C. frutescens*	12	2	14

### Growth Conditions and Phenotyping

Five seeds of each line were sown either in the field or greenhouse at Seoul National University (Suwon, South Korea). MJ was grown in the greenhouse during 2017 and in the open field in 2018; FC and PD were grown in greenhouses for two consecutive years, (2016/2017 and 2017/2018, respectively); and the UA and LA populations were grown once in 2018 and 2019, respectively, in greenhouses. The core collection was planted in a greenhouse owned by Biotong Seed Co. Ltd., Anseong, South Korea, in 2018. The F_1_ and F_2_ generations of the biparental lines were used for an allelism test, and data on fruit orientation was recorded from all plants included in the experiment. Quantitative traits included in this experiment were measured for five representative fruit samples of each plant. The lengths and widths of the pedicels and fruits were measured using calipers, and the fresh weight of the fruits was determined using a digital balance.

### Light Microscopic Observation

Light microscopic analysis was used to observe the cross-sectional and longitudinal part of the pedicel at the point of curvature for pendent types and at near attachment point to the fruit for straight types. The cut part was stained with 0.05% toluidine blue O in 2.5% sodium carbonate solution and semi-thin sections were observed and photographed using an Axiophot photomicroscope (Zeiss) as described previously (Jeong et al., 2014).

### Genomic DNA Extraction

Two to three young leaves from each plant were subjected to DNA extraction using the cetyltrimethylammonium bromide (CTAB) method. Leaf tissues were ground using a TissueLyser II (Qiagen, Hilden, Germany). The concentration and purity of the DNA samples was measured using a NanoDrop (Thermo Fisher Scientific, Waltham, MA, United States). DNA samples showing absorbance ratios at 260/280 nm above 1.8 were diluted to a final concentration of 50 ng/μL with distilled water for downstream analysis.

### Genotyping-by-Sequencing (GBS)

Genotyping-by-sequencing libraries were constructed from the DNAs of the FC and MJ populations based on the *Pst*I*/Mse*I and *Eco*RI*/Mse*I restriction enzymes (Han et al., 2018). Pooled libraries were sequenced using an Illumina HiSeq2000 sequencing system (Illumina, San Diego, CA, United States) at Macrogen (Seoul, South Korea).

### Development of SNP Markers and Linkage Analysis of Molecular Markers

The reanalysis of the PD sequencing data (Han et al., 2018) and the GBS analysis of the FC and MJ populations were performed using the updated genome reference for *C. annuum* cv. CM334 version 1.6^1^ (Kim et al., 2014), ‘‘L_Zunla-1’’^2^ (Qin et al., 2014), and the newly developed ‘Dempsey’ (unpublished data of our laboratory) reference genomes. Use of different reference genomes provided additional advantages in narrowing the target region by correcting some sequencing errors due to the limitation of the short-read sequencing technology. Quality control and GBS sequence data trimming were performed using the CLC Genomics Workbench version 6.5 (Qiagen) using the settings Q20 and a minimum read length of 30 bp. The trimmed sequence reads were mapped to each of the reference genomes using Burrows–Wheeler Aligner version 0.7.12 (Li et al., 2013). Picard Tools version 1.119 and SAMtools version 1.1 were used for read grouping and sorting (Li et al., 2009). For genome-wide SNP calling, Genome Analysis Toolkit Unified Genotyper version 3.3 was used. High-quality SNPs with a quality value over 30 and a minimum depth of 3 were selected for further analysis. A bin linkage map was constructed, and a sliding window approach was used to impute missing data and genotyping errors, as described previously (Han et al., 2016). Windows QTL Cartographer 2.5 was used for the analysis of possible fruit orientation–related QTLs in the PD, FC, and MJ populations using composite interval mapping using the default parameters (Zeng, 1994). The threshold LOD scores were calculated using 1,000 permutations with a significance level of 0.05 and the loci with LOD value higher than the threshold were considered as QTL. Major QTL was defined as QTL scores *R*^2^ ≥ 20%. The additive effect and the proportion of the observed phenotypic variation (*R*^2^) for each QTL were also obtained using same software. Dominance effects for PD and FC populations were not estimated as they are RIL populations. Any QTL within 10 cM distance on chromosome 12 were regarded as a single QTL.

Settlement of MLM Under Progressively Exclusive Relationship (SUPER) GWAS was utilized (Wang et al., 2014) by the R package Genomic Association and Prediction Integrated Tool (GAPIT), using default parameters (Lipka et al., 2012). The R scripts used for GWAS is attached in the Supplementary File (script_SUPERgwas). All the probabilities generated in the association runs were transformed using log10P(0.05), as described previously (Siddique et al., 2019). The scores for chromosome 12 were then inspected in Manhattan plots to determine whether the SNPs reached the significance threshold. The −log10P values of SNPs from the GWAS were adjusted using a Bonferroni multiple test correction.

Genotyping of individual markers was performed using a high-resolution melting (HRM) analysis as described by Park et al. (2009). HRM was carried out in 20-μL reaction mixtures on a Rotor-Gene 6000 thermocycler (Corbett; Qiagen). Each reaction contained 10 (PCR reaction buffer, 2.5 mM of each dNTP, 10 pmol of each primer, 0.2 U Taq polymerase, 1.25 μM Syto9 (Thermo Fisher Scientific), and 50 ng gDNA. The thermocycling conditions were 95°C for 5 min, followed by 50 cycles of 95°C for 20 s, 58°C for 20 s, and elongation at 72°C for 20 s. HRM marker analysis was carried out denaturation at 90°C for 10 min, followed by 50 cycles of 96°C for 20 s, 57°C for 20 s, and 72°C for 40 s. Holding temperatures of 95°C and 40°C for 1 min were added. HRM was analyzed with increasing temperature 0.1°C every minute from 65°C to 95°C.

### PCR Amplification and Localization of the *CapUp* Gene

The region containing the *CapUp* locus was amplified using a polymerase chain reaction (PCR), performed using a 50-μL reaction mixture containing 50 ng template DNA (2 μL), 10 × PCR buffer, 2.5 mM of each dNTP mix, 10 pmol/μL of each primer, and 1 U Taq DNA polymerase (Takara Korea Biomedical Inc., Seoul, South Korea). The PCR cycling program was as follows: 95°C for 5 min; 35 cycles of 95°C for 30 s, 58°C for 30 s, and 72°C for 1 min 30 s; and a final step of 72°C for 10 min. The PCR products were analyzed on a 1% agarose gel in 1 × TAE buffer and visualized using a Bio-Rad Universal Hood II Gel Doc System (Bio-Rad Laboratories, Hercules, CA, United States) after staining with ethidium bromide.

Polymerase chain reaction primers were designed using Primer3 software^3^ (Rozen and Skaletsky, 2000). A co-segregation analysis of the pheno-genotypes enabled the development of closer SNP markers that could be used in the FC, UG, and LA populations. Polymorphic markers that co-segregated with the phenotype were combined for the linkage analysis and used to develop the genetic linkage map.

### Gene Sequencing

To confirm the SNPs and identify the nucleotide variation between the parental lines at these locations, a sequence analysis was performed using the PCR products obtained from plants found to contain different alleles for the fruit orientation. The PCR products were identified on 1% agarose gels using electrophoresis. The PCR products were purified using a Gel and PCR Clean-up kit (Cosmo Genetech, Seoul, South Korea). Sanger sequencing was conducted at Macrogen, and the DNA sequences were analyzed using the Lasergene SeqMan program (DNASTAR, Madison, WI, United States).

### Real-Time Quantitative PCR (qRT-PCR)

Total RNA was extracted from the pedicels of homozygous pendent and erect inflorescence buds (before flower blooming) using MG RNAzol Kit (MGmed, Seoul, South Korea), according to the manufacturer’s instructions. Expression levels of genes putatively associated with fruit orientation were analyzed using a Lightcycler 480 Real-Time PCR system (Roche, Basel, Switzerland). The qRT-PCR was performed using the following conditions: 95°C for 5 min, followed by 45 cycles of 95°C for 10 s, 60°C for 20 s, and 72°C for 20 s. The expression levels of the candidate genes were calculated relative to the reference gene *CaActin*.

### Semi-Quantitative RT-PCR

The expression levels of *EFL3-1* and *EFL3-2* in pedicel of ‘UB7’ and ‘GB57’ were measured by semi-quantitative RT-PCR. RT-PCR was conducted as following conditions: initial denaturation at 95°C for 5 min, 20, 25, and 28 cycles of denaturation at 95°C for 10 s, annealing at 58°C for 20 s, and extension at 72°C for 20 s. Relative expression levels of *EFL3-1* and *EFL3-2* in ‘UB7’ and ‘GB57’ were normalized to *CaActin* expression.

## Results

### Fruit Orientation in Pepper and Its Temporal Change

**FIGURE 1:**
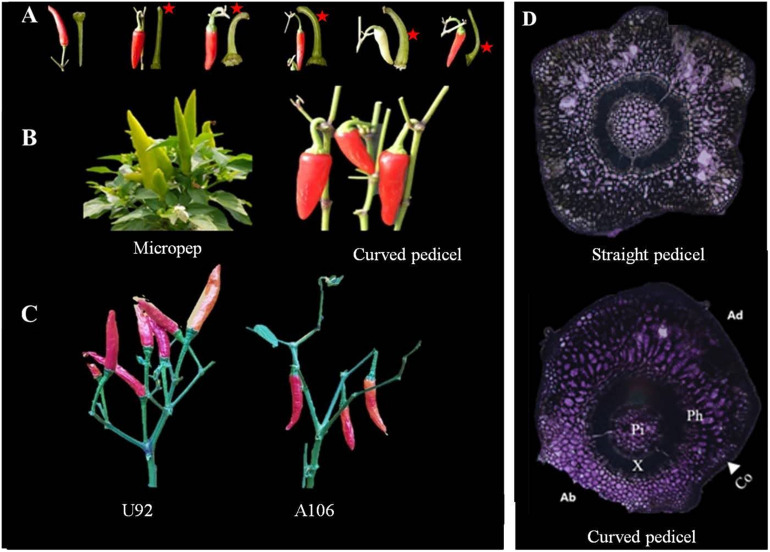
Pedicel curvature and fruit orientation phenotype. **(A)** Pedicels with different positions of curving, marked with red asterisks. **(B,C)** Vertically upright (left) *Capsicum annuum* ‘micropep’, *C. annuum* ‘U92’ and vertically pendent (right) *C. annuum* ‘Jeju,’ *C. annuum* ‘A106’ fruit types. **(D)** Cross-section view of an erect (straight) pedicel with a uniform distribution of cells across the surface (top) and a pendent pedicel at the point of curvature (bottom), where the abaxial cells (Ab) are on average 2.6× more compacted than the cells on the adaxial side (Ad). Co, collenchyma; Ph, phloem; X, xylem; Pi, pith.

Fruit orientation, which is mainly governed by the curvature of the pedicels, may vary depending on the relative position at which the bending occurs on the pedicel: near the point of attachment with the branch or further away toward the fruit (Figure 1A). Those plants with curvature at the base of the pedicel, which was observed in all parental pendent lines used in the experiment (Figures 1B,C), were strictly found to be pendent types, with no confusing phenotypes even during the early flowering period. For some lines in the segregating populations, however, such as in LA F_2_, the fruit orientation could not be determined during the flowering and early fruiting stages as the phenotypes can shift from one state to the other, perhaps due to variation in the weather or the growing stage. In others, commonly in the GWAS population, however, lateral pendent (horizontally oriented) and lateral erect fruits were observed not only because of the curvature of the pedicels, but also because of the overall loose architecture of the plant, the branch growth habit, fruit weight, and pedicel length (Supplementary Figure 1). Accordingly, loose-branched peppers with long pedicels and heavy fruits tend to grow horizontally or become pendent, even though the pedicels are erect.

### Pedicel Morphology in a Segregating Population and Its Correlation With Other Related Traits

To elucidate pedicel morphology and its correlation with some fruit-related traits in the orientation of fruit growth, we observed representative samples from PD in 2017 and MJ in 2019. Generally, the average pedicel length was higher for PD than MJ, with respective values of 3.4 cm (range 1.8–6.5 cm) and 2.9 cm (range 1.9–5.8 cm). There was no difference in the average pedicel thickness between the two populations, with both averaging 0.4 cm, while the majority (80%) were between 0.35 and 0.45 cm in diameter. The average pedicel length was invariably longer for pendent-oriented types than the lines with upright fruits in both populations, while there was no variation in pedicel thickness for both types.

The straight and curved pedicels of the MJ parental lines were analyzed using a light microscope by horizontal-sectioning mature tissues. In the curved pedicels, the abaxial collenchyma cells around the curvature of the pedicels are smaller and denser than the collenchyma cells on the adaxial side, while other components of the internal tissue (pith, xylem, and phloem) were unaffected (Figure 1D). In the straight pedicels, there was no variation in the cellular number or size of any of the components. A Pearson correlation matrix generated for the PD population showed a positive correlation between fruit weight, pedicel length, and pendent orientation (Supplementary Table 1 and Supplementary Figure 2). A negative non-significant correlation was also observed between the length and thickness of the pedicels, and between the pedicel thickness and pendent fruit orientation.

### Inheritance Analysis of Fruit Position

Two intraspecific segregating populations were analyzed to understand whether the fruit orientation is similar among different populations of pepper, to study its inheritance, and to confirm what has been reported previously (Lee et al., 2008, 2016; Cheng et al., 2016). The *C. annuum* parental lines A79 and Jeju showed a pendent fruit orientation, whereas LP97 and Micropep produce erect fruit. Although fruit orientation appears to be a qualitative trait in this study, we observed four types of orientation; vertical upright, in which all fruits are held vertically in an erect position; vertical pendent, where all fruits are vertically pendent; lateral pendent, where the majority of fruit have pendent growth with some horizontal orientation; and lateral erect, in which the majority of fruit are held in a vertically erect position with some horizontal growth (Figure 1C and Supplementary Figures 1A,B). For the inheritance study, we considered the lateral pendent group as pendent and lateral erect group as erect.

Accordingly, of the 379 F_2_ plants from the LA population, 291 were pendent and 88 were erect, fitting a 3:1 ratio (χ^2^ = 0.64, *p* = 0.50) for a single dominant gene (Table 2). Similarly, of the 214 F_2_ plants of MJ, 154 showed a pendent phenotype and the remaining 60 were erect, following the Mendelian segregation ratio (3:1, χ^2^ = 1.05, *p* = 0.30). These results suggests that the pendent fruit orientation in pepper is dominant over erect types.

**TABLE 2:** Segregation analysis of fruit orientation for the LA and MJ populations.

Population	Generation	No. of plants	Phenotype		Expected ratio		
			Pendent	Erect	Pendent:Erect	χ^2^	*P*-value
A79	Parent	20	20	–	20:0		
LP97	Parent	20	–	20	0:20		
LA	F_2_	379	291	88	3:1	0.64	0.50
MJ	F_2_	214	154	60	3:1	1.05	0.30

*A79 and LP97 are the parental lines of the LA population; MJ is a population derived from the Micropep and Jeju parental lines.*

Fruit orientation was also segregated in the four *Capsicum* species of the core collection included in this experiment. There were 57, four, one, and 12 accessions with upright fruit positions out of a total of 196, 25, 21, and 14 germplasms of *C. annuum, C. baccatum, C. chinense*, and *C. frutescens*, respectively (Table 1).

### Localization of the *CapUp* Locus

Considering the lateral pendent and lateral erect phenotypes as pendent and erect, respectively, our inheritance study suggested that fruit orientation is controlled by one major gene; however, fruit orientation is not strictly qualitative, as was described by Cheng et al. (2016). We therefore decided to perform an analysis of the responsible loci using a QTL approach on different biparental populations and a SUPER GWAS using the diversity panel, with the aim of identifying a colocalized region before fine mapping the gene.

**FIGURE 2:**
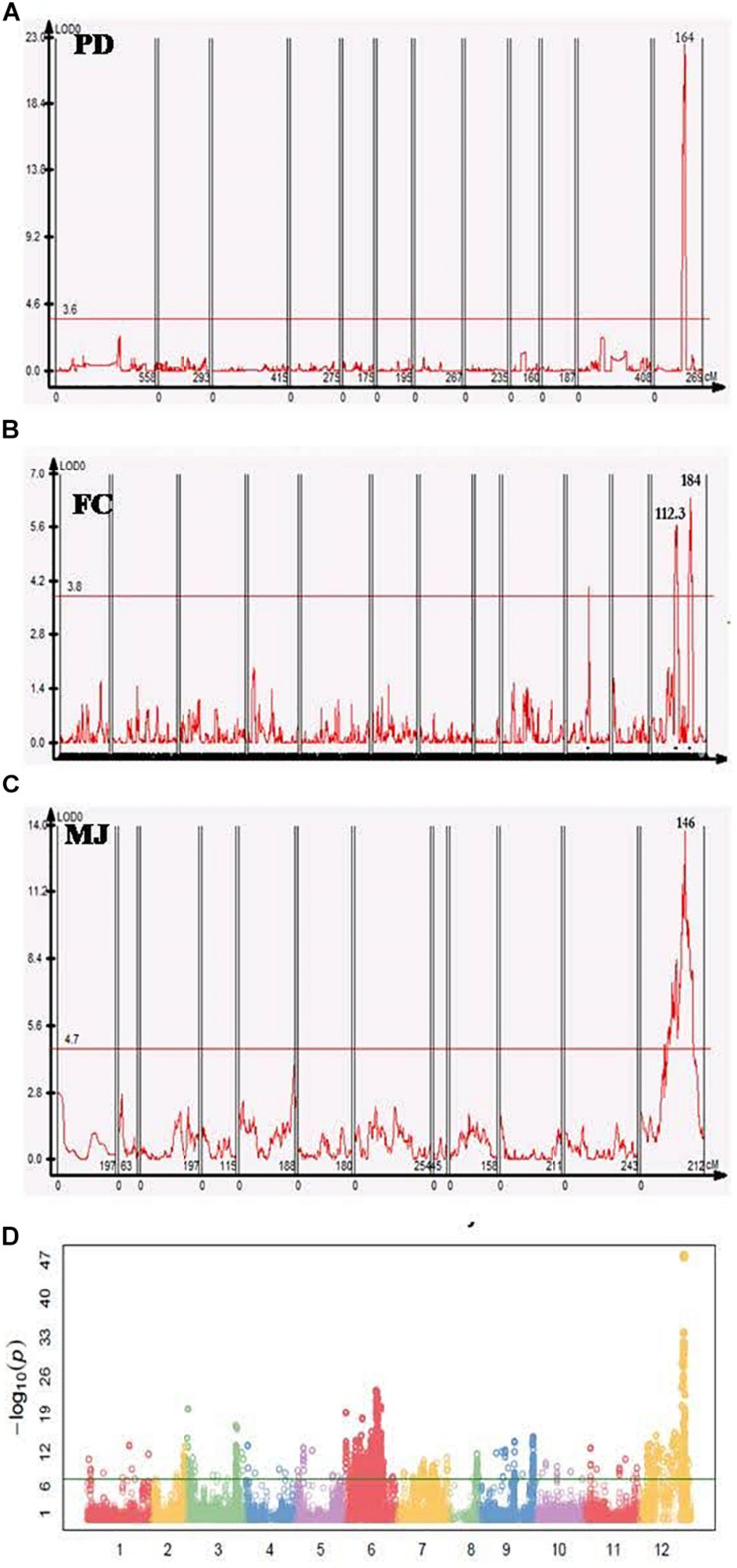
Genome-wide association study (GWAS) and QTL analyses of fruit orientation in different populations. **(A–C)** Fruit orientation QTLs in the PD, FC, and MJ populations, all of which have similar significant QTL regions on chromosome 12. One peak was present in PD, two peaks were detected in FC, and one peak was detected in MJ. **(D)** Manhattan plot of the core collection, showing a significant peak on chromosome 12 (–LogP of 47) associated with fruit orientation.

In the PD population a significant QTL with a log-likelihood (LOD) score of 20.9 was detected on chromosome 12, that explained 49.1% of the phenotypic variation. The QTL region was mapped at 165.8–168.7 cM- an interval of 5.3 cM- between two markers that were physically located in the region of 203–208 Mb (∼5 Mb) (Figure 2A). In the FC population, there were two minor QTL with LOD values of 5.7 and 6.4, that detected high phenotypic variation explained (PVE) of 13.8% and 15.4%, respectively (Figure 2B). Three markers were located closest to these QTL peaks, spanning 113.4–180.6 cM with a corresponding physical distance of 97.6–229.8 Mbp in the same chromosome. One major QTL on chromosome 12 was detected in MJ between 57.7 and 242 Mbp, explaining 8.1% of the phenotypic variation (*R*^2^) with an LOD value of 11.6 (Figure 2C and Table 3).

**TABLE 3:** Detected QTL regions associated with fruit orientation in the three-selected populations.

Population	Chromosome	QTL position (cM)	LOD	Coverage (cM)	Additive	Dominance	PVE (%)	*R*^2^
PD	12	165.81	20.9	5.3	0.50	ND	58.69	49.1
FC	12	113.41	5.7	9	−0.18	ND	13.78	9.6
FC	12	180.61	6.4	3.3	−0.2	ND	15.39	10.5
MJ	12	138.51	11.6	17.5	0.03	−0.65	50.83	8.1

*QTL, Quantitative Trait Loci; LOD, logarithms of the odds; PVE, phenotypic variation explained; R^2^, coefficient of determination. ND, not determined.*

In addition to the QTL mapping, we used SNP data for the pepper core collection (Lee et al., 2016) of 256 plants (Table 1) to conduct a GWAS for fruit orientation. A minor allele frequency of >0.05, SNP coverage of >0.6, and inbreeding coefficient >0.8 were used as a filtering criteria to obtain 176,951 high-quality SNPs for the downstream analysis. Excluding all false positive results below the Bonferroni correction, we identified 14 highly significant SNPs associated with fruit orientation between 205 and 214 Mbp on chromosome 12 (Figure 2D).

### Fine-Mapping of the *CapUp* Locus and Validation of Markers

**FIGURE 3:**
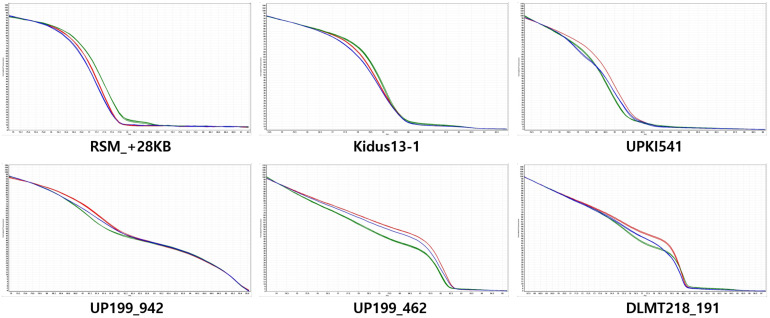
Normalized codominant high-resolution melting (HRM) curves for fruit orientation in the LA F_2_ population. The *y*-axis shows temperature and the *x*-axis shows normalized fluorescence. Six HRM markers are codominant predictors of the genotype of the dominant pendent (green), recessive erect (red), and heterozygous (blue) types. The UP199_942 and UP199_462 markers are completely linked to the fruit orientation trait (0 cM).

From the QTL mapping of PD, FC and MJ and the GWAS analysis of the core collection results, the *CapUp* locus was found to be located between 200 and 250 Mbp on chromosome 12. Primers were designed to amplify fragments within the mapped *CapUp* region using Dempsey version 1.0 reference genome (unpublished). The amplified fragments were sequenced and the SNPs between parents were identified. Based on these SNPs, HRM markers were developed to fine map the fruit orientation locus (Supplementary Table 2). The six developed HRM markers (DLMT218_191, UP199_462, UP199_942, UPKI541, Kidus13-1, and RSM_+28KB) were used to show clear patterns of dominant homozygous, heterozygous, and recessive homozygous genotypes (Figure 3). The six markers were analyzed to fine map the *CapUp* locus using 335 plants from the LA F_2_ population of 379 plants.

**FIGURE 4:**
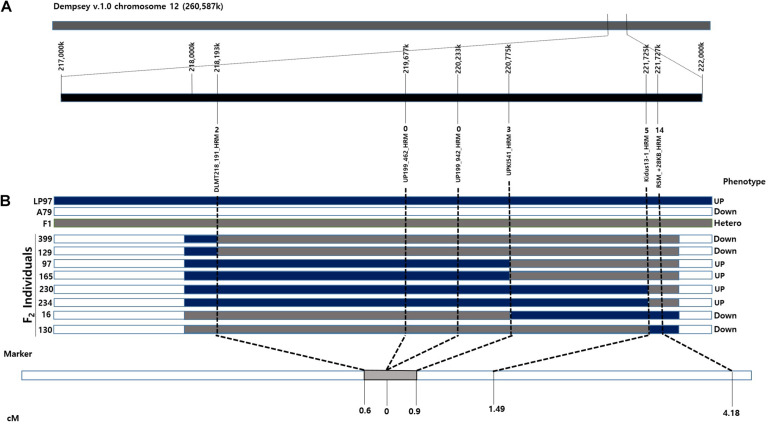
Synteny between the genetic and physical maps at the *CapUp* region in the LA F_2_ population. **(A)** The candidate *CapUp* region is located between 218 and 222 Mbp on chromosome 12. LP97 (blue) is homozygous recessive, F_1_ (gray) is heterozygous, and A79 (white) is homozygous dominant. The names of the HRM markers are indicated above the horizontal black line. Recombinant plants are indicated above the HRM markers. **(B)** Genetic location of the *CapUp* locus in the LA F_2_ population. The *CapUp* locus was mapped between DLMT218_191 and UPKI541, which are located at 0.6 and 0.9 cM, respectively.

Based on the fine mapping results, the DLMT218_191 marker was found to be located in the region of 218 Mbp along chromosome 12 and the RSM_+28KB marker was located at 221 Mbp, with a physical distance of 3.58 Mbp and a genetic distance of 4.18 cM between them. Primers were then designed to narrow the candidate *CapUp* region. After sequencing the primer target regions, four markers were developed using SNPs between the parent plants LP97 and A79. Finally, the candidate *CapUp* region was delimited between DLMT218_191 and UPKI541, and the delimitation region interval was narrowed down to 2.58 Mbp. Two markers DLMT218_191 and UPKI541 showed two and three recombinants among 335 LA-F_2_ individuals. The genetic distance of two markers were 0.6 and 0.9 cM from the *CapUp* locus, respectively. Two markers (UP199_462 and UP199_942) were found to be completely linked to *CapUp* (Figure 4). When using the UP199_942 marker, cosegregation was observed in 98 UG-F_2_ individuals. But polymorphism was not found in FC population. By contrast, cosegregation was observed using UP199_462 marker in FC and UG populations.

### Expression Analysis of Candidate Genes and Sequence Variation of Candidate Genes

**FIGURE 5:**
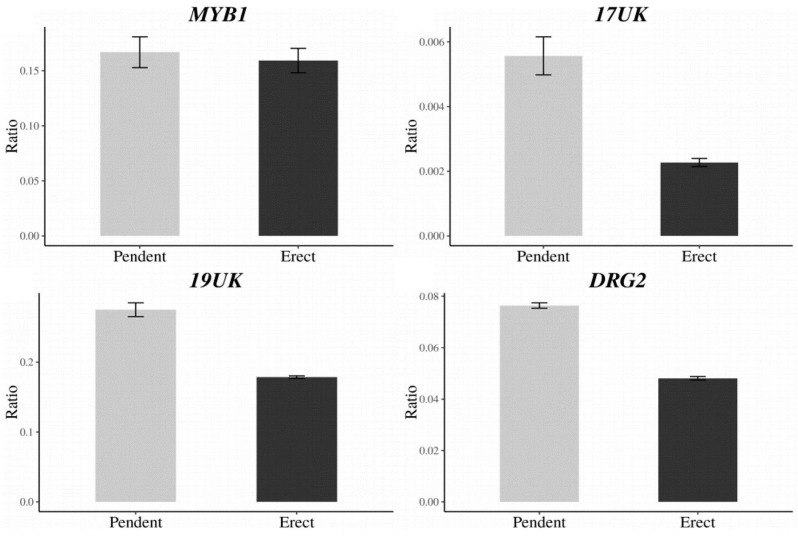
Expression levels of the *CapUp* candidate genes (*MYB1*, *17UK*, *19UK*, and *DRG2*) in UG population. The *y*-axis shows the expression level relative to that of the housekeeping gene *Actin*. The x-axis shows phenotype. The bars indicate the range of standard error of the mean.

A total of 27 candidate genes were identified within the delimited region between DLMT218_191 and UPKI541. Among these genes, seven candidate genes for *CapUp* were located between UP199_462 and UP199_942 markers (Table 4). To analyze the difference in the expression levels of these genes between plants showing different fruit orientations, a qRT-PCR was performed for the six genes [since there were two copies of the *ELF4-LIKE 3* (*EFL3*) gene] in UG population. A qRT-PCR and semi-quantitative RT-PCR analyses showed that there were significant differences in the expression levels of three genes, *17UK* (17 unknown protein), *19UK* (19 unknown protein), and *DRG2* (*Developmentally-regulated G protein 2*), whereas there was no difference in expression levels of *MYB1* (*MYB transcription factor 1*), *EFL3-1*, and *EFL3-2* (Figure 5 and Supplementary Figure 3).

**TABLE 4:** Candidate genes in the *CapUp* mapping region.

Gene ID	Chr.	Position	Description
DEM.v1.00034670	12	218247943–218248991	1 Protein of unknown function
DEM.v1.00034671	12	218282291–218283246	2 Protein of unknown function
DEM.v1.00034675	12	218302416–218302787	3 Protein of unknown function
DEM.v1.00034676	12	218309056–218310648	*At1g17410: Probable nucleoside diphosphate kinase 5 (Arabidopsis thaliana)*
DEM.v1.00034682	12	218691993–218692298	5 Protein of unknown function
DEM.v1.00034683	12	218801228–218801644	6 Protein of unknown function
DEM.v1.00034684	12	219040631–219043650	*RIPK: Serine/threonine-protein kinase RIPK (Arabidopsis thaliana)*
DEM.v1.00034685	12	219115798–219116076	8 Protein of unknown function
DEM.v1.00034686	12	219158720–219159133	9 Protein of unknown function
DEM.v1.00034687	12	219206551–219207412	*PBL13: Serine/threonine-protein kinase PBL13 (Arabidopsis thaliana)*
DEM.v1.00034689	12	219318978–219319447	11 Protein of unknown function
DEM.v1.00034691	12	219400771–219412417	*SPL2: E3 ubiquitin-protein ligase SPL2 (Arabidopsis thaliana)*
DEM.v1.00034692	12	219413527–219414412	*NSP1: Protein NODULATION SIGNALING PATHWAY 1 (Medicago truncatula)*
**DEM.v1.00034696**	**12**	**219745447–219746073**	***ABP19A: Auxin-binding protein ABP19a (Prunus persica)***
**DEM.v1.00034699**	**12**	**219786160–219788335**	***MYB1: Transcription factor MYB1 (Crocosmia* × *crocosmiiflora)***
**DEM.v1.00034700**	**12**	**219892613–219893059**	***EFL3: Protein ELF4-LIKE 3 (Arabidopsis thaliana)***
**DEM.v1.00034701**	**12**	**219894021–219896479**	**17 Protein of unknown function**
**DEM.v1.00034702**	**12**	**219989967–219990413**	***EFL3: Protein ELF4-LIKE 3 (Arabidopsis thaliana)***
**DEM.v1.00034703**	**12**	**219991340–219996497**	**19 Protein of unknown function**
**DEM.v1.00034706**	**12**	**220027552–220035732**	***DRG2: Developmentally-regulated G-protein 2 (Arabidopsis thaliana)***
DEM.v1.00034718	12	220250483–220253688	*28 kDa ribonucleoprotein chloroplastic (Nicotiana sylvestris)*
DEM.v1.00034720	12	220336214–220336639	22 Protein of unknown function
DEM.v1.00034723	12	220442614–220445278	23 Protein of unknown function
DEM.v1.00034725	12	220539715–220540107	24 Protein of unknown function
DEM.v1.00034726	12	220651134–220652283	*Protein BIG GRAIN 1-like A (Arabidopsis thaliana)*
DEM.v1.00034727	12	220657333–220674748	*GIP1: GBF-interacting protein 1 (Arabidopsis thaliana)*
DEM.v1.00034728	12	220693721–220705876	27 Protein of unknown function

*Based on Dempsey version 1.0 annotation data. Bolded terms: Candidate genes for CapUp between UP199_462 and UP199_942 markers.*

To identify any differences in the coding sequences of the candidate genes, the exon regions of each gene were analyzed. There were no differences in the *EFL3*, *17UK, 19UK*, or *DRG2* sequences, while two nucleotide differences were detected in *MYB1* at the 75th nucleotide [G (pendent) to A (erect)] and the 553th nucleotide [T (pendent) to C (erect)] [Supplementary Figures S4–S9 and Supplementary File (gene sequence files)]. Both nucleotide changes resulted in missense mutations in the amino acid sequence. We were unable to examine any potential sequence differences in *ABP19A* (*Auxin-binding protein ABP19a*) due to the very high copy number of the gene.

## Discussion

This study was designed to fine map the genome region controlling fruit orientation in *Capsicum* and identify the responsible candidate genes using segregating populations and diverse accessions, employing a combination of QTL and GWAS analysis.

Pepper pedicel curvature typically determines fruit orientation (Sun et al., 2019). The relative position at which the bending occurs on the pedicel can result in either a vertical pendent phenotype, which is easy to distinguish even at early flowering stages and is characteristic of certain populations, or a lateral pendent phenotype, possessing an almost horizontally oriented fruit tip (Cheng et al., 2016). In some populations, it was difficult to determine whether the final fruit orientation would be upright or pendent during the flowering and early fruiting stages because the phenotypes can shift from one state to the other. Similar observations of *C. annuum* genotypes were also reported by Munting (1974).

The inheritance of the fruit orientation trait in *Capsicum* was investigated by crossing pendent- and upright-fruited parental lines. After categorizing the two intermediate types of fruit orientation into the main two types, our results suggested that the pendent fruit orientation in pepper is dominant over the erect form. Similarly, Cheng et al. (2016) reported that the erect phenotype is controlled by a recessive gene with incomplete inheritance.

Understanding the relationships between pepper fruit orientation and some fruit-related traits is very important because a change in one character can fully or partially influence the other one. Our identification of a positive correlation between fruit weight and pedicel length contributing to the tendency of pendent growth is consistent with previous reports from Han et al. (2016). Since the fruits are set at the tip of a non-wooden pedicel, it is theoretically logical that these stalks would bend downward as the length of the fruit-bearing pedicel increased. We observed a negative correlation between pedicel thickness and length, and between pendent growth and pedicel thickness. The negative correlation between the length and thickness of the pedicel can be explained by the similar tissue volume of pedicels in the two types, which can also be substantiated by the observation we made regarding the reduced size of the collenchyma cells in the abaxial pedicel region of the pendent types. This might explain the negative correlation between pedicel thickness and pendent fruit growth.

To understand the trend of fruit orientation in different pepper populations, identify the possible minor alleles that contribute to the few intermediate phenotypes, and determine the major locus controlling upright and pendent fruit orientations, we performed QTL and GWAS analyses. The combined use of QTLs and GWAS was previously shown to be a powerful approach for the identification of loci and candidate genes in pepper (Han et al., 2018), as well as other crops (He et al., 2017; Liu et al., 2018; Zhao et al., 2018; Bo et al., 2019; Siddique et al., 2019).

Using a composite interval mapping approach with three biparental populations, our mapping region was delimited to 200–250 Mbp on chromosome 12. The first molecular study to develop amplified fragment polymorphic and CAPS markers for the elucidation of the pendent orientation of *C. annuum* using 108 *F*_2__:__3_ individuals also showed the gene responsible was located on chromosome 12 (Lee et al., 2008). Han et al. (2016) identified two consistent QTL regions in the same chromosome (*FP-12.1* and *FP-12.2*) for fruit position in the PD RIL grown in two different environments. Of the two QTLs, they reported that *FP-12.2*, located at 199.6 Mbp of the CM334 version 1.55 reference genome, was the possible locus containing the major fruit orientation gene, as this region explained over 40% of the phenotypic variation. In the same year, a major QTL named Up12.1 was detected in the same region using 297 F_2_ lines obtained from the interspecific cross of *C. annuum* BA3 and *C. frutescens* YNXML (Cheng et al., 2016).

The pepper core collection (Lee et al., 2016) was used for our SUPER GWAS, in which we identified 14 SNPs that were highly significantly associated with fruit orientation in the physical position 205–214 Mbp in chromosome 12 with a −log10(*p*) value > 26 (Wang et al., 2014). By combining these results and previously published reports (Lee et al., 2008; Cheng et al., 2016; Han et al., 2016), we identified a target region between 218 and 222 Mbp (Dempsey version 1.0 reference genome) for further study.

We used six newly developed markers and selected different HRM markers to narrow the genomic region containing the gene controlling fruit orientation to 2.58 Mbp on chromosome 12, which was delimited by two flanking HRM markers, DLMT218_191 and UPKI541. These markers were found to be 0.6 and 0.9 cM from the gene, respectively. Furthermore, the new UP199_462 and UP199_942 markers were completely linked to the erect phenotype, which will assist the future selection of peppers with this fruit orientation.

A strong candidate gene for *CapUp* would be related to cell development and proliferation. Among the candidate genes, *MYB1* and *DRG2* were selected as the strongest candidates. Upon further analysis, no difference was detected in the expression levels of *MYB1* between the pendent and erect plants, although the identified amino acid sequence change (E to G) altered the polarity of the sequence, which might affect the folding of the protein. Furthermore, MYB proteins are key factors in the networks regulating plant development, metabolism, and responses to biotic and abiotic stresses (Dubos et al., 2010). By contrast, there was no difference in the nucleotide sequence of *DRG2* between the pendent and erect plants, but we did identify a difference in the expression level of this gene. Plant G proteins are also involved in the regulation of almost every aspect of growth, development, the responses to environmental and hormonal signals, and the responses to biotic and abiotic stresses, as well as the control of cell division and the regulation of ion channel activity (Pandey and Vijayakumar, 2018). The loss-of-function G protein mutants have altered auxin-mediated cell division throughout their development (Ullah et al., 2003). To elucidate why *DRG2* is differentially expressed in the pendent and erect plants, further studies should examine whether it contains variations in its promoter sequence. *ABP19A* is an AUXILIN-like protein and another candidate gene for *CapUp*, although we were unable to detect sequence and expression variations for this gene due to its high copy number [Chromosome 3, 5 (two copies), and 12]. In Arabidopsis, there are seven AUXILIN-like proteins, named AUXILIN-LIKE 1–7, in addition to AUXIN-LIKE 1. AUXILIN-LIKE 1 and AUXILIN-LIKE 2 are clathrin uncoating factors involved in clathrin-mediated endocytosis. Clathrin-mediated endocytosis (CME) is a cellular trafficking process, in which cargoes and lipids are internalized from the plasma membrane into vesicles coated with clathrin and adaptor proteins. CME is essential for many developmental and physiological processes in plants. Adamowski et al. (2018) searched for new factors in CME in *A. thaliana* by performing tandem affinity purification of proteins that interact with clathrin light chain, a principal component of the clathrin coat. In addition, they found that two putative homologs of the clathrin-coat uncoating factor auxilin. Overexpression of AUXILIN-LIKE1 and AUXILIN-LIKE2 in Arabidopsis caused an arrest of seedling growth and development (Adamowski et al., 2018; Sun et al., 2019).

Further functional studies of these potential candidate genes should be conducted to elucidate the molecular mechanisms underlying fruit orientation in pepper. The findings and markers developed in this study will be helpful in pepper breeding.

## Data Availability Statement

Datasets generated for this paper can be found in The National Agricultural Biotechnology Information Center (http://nabic.rda.go.kr/). Please refer to the information below for a detailed link. (1) DLMT218_191, http://nabic.rda.go.kr/nolog/NP-1412-000001/snpView.do; (2) Kidus13-1, http://nabic.rda.go.kr/nolog/NP-1413-000001/snpView.do; (3) RSM_+28KB, http://nabic.rda.go.kr/nolog/NP-1414-000001/snpView.do; (4) UP199_462, http://nabic.rda.go.kr/nolog/NP-1415-000001/snpView.do; (5) UP199_942, http://nabic.rda.go.kr/nolog/NP-1416-000001/snpView.do; (6) UPKI541, http://nabic.rda.go.kr/nolog/NP-1417-000001/snpView.do.

## Author Contributions

AS and T-GK contributed to the experiments and writing of the manuscript. AS conducted phenotyping in *up* gene populations and QTL mapping. T-GK conducted fine mapping, qRT-PCR, and identification of gene variations. KH constructed PD RILs and contributed GBS experiments. H-YL constructed core collection of pepper and conducted genotyping in the core collection. AP and MS assisted with GWAS analysis and QTL mapping. JA provided parental lines and UG-F_2_ population. B-CK supervised the entire process. All authors contributed to the article and approved the submitted version.

## Conflict of Interest

The authors declare that the research was conducted in the absence of any commercial or financial relationships that could be construed as a potential conflict of interest.

## Footnotes

1 http://peppergenome.snu.ac.kr/

2 http://peppersequence.genomics.cn

3 https://bioinfo.ut.ee/primer3-0.4.0/

## Supplementary Material

Supplementary Figure 1 Intermediate fruit orientation phenotypes. **(A)** Lateral pendent phenotype. **(B)** Lateral upright phenotype.

Supplementary Figure 2 Frequency distribution of traits related to fruit orientation. Pedicel length, pedicel thickness (width), and fruit weight of the PD and MJ F_2_ populations associated with the erect and pendent fruit orientations.

Supplementary Figure 3 Expression analysis of *EFL3-1* and *EFL3-2* in UB7 (erect) and GB57 (pendent). **(A,B)** In the pedicel of ‘UB7 and GB57’, *EFL3-1, EFL3-2*, and *CaActin* were not expressed. **(C)** *EFL3-1* and *EFL3-2* expression levels were identified.

Supplementary Figure 4 Sequence variation analysis of *MYB1*. **(A)** Nucleotide sequence alignment. **(B)** Amino acid sequence alignment.

Supplementary Figure 5 Alignment of all *EFL3-1* exon sequences.

Supplementary Figure 6 Alignment of all *17UK* exon sequences.

Supplementary Figure 7 Alignment of all *EFL3-2* exon sequences.

Supplementary Figure 8 Alignment of all *19UK* exon sequences.

Supplementary Figure 9 Alignment of all *DRG2* exon sequences.

Supplementary Table 1 Pearson correlation matrix of fruit orientation–related traits in the PD lines.

Supplementary Table 2 Primers used in this study.
