# Supplementary figures and images for: Fine Mapping and Candidate Gene Identification for the CapUp Locus Controlling Fruit Orientation in Pepper (Capsicum spp.)
Source: Front Plant Sci. 2021 Jun 28;12:675474. doi: 10.3389/fpls.2021.675474 (PMC8273576; doi:10.3389/fpls.2021.675474)

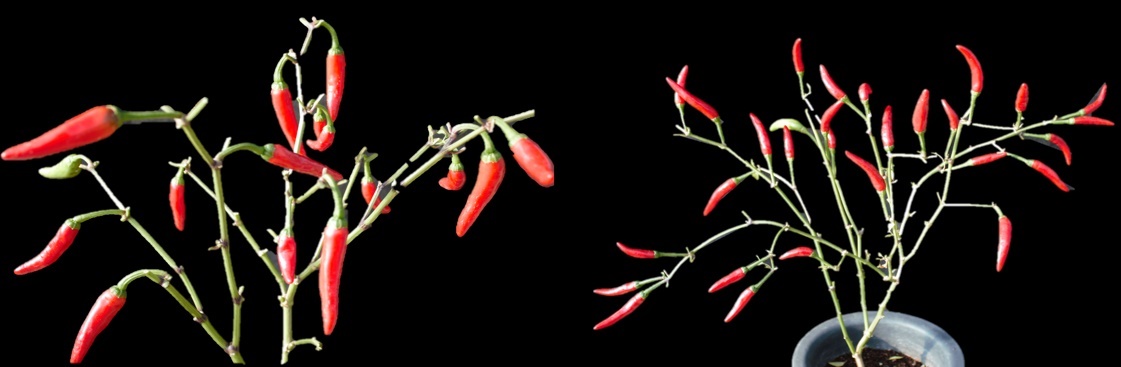

Supplement: Supplementary Figure 1 — Intermediate fruit orientation phenotypes. (A) Lateral pendent phenotype. (B) Lateral upright phenotype. [file Image_1.JPEG]

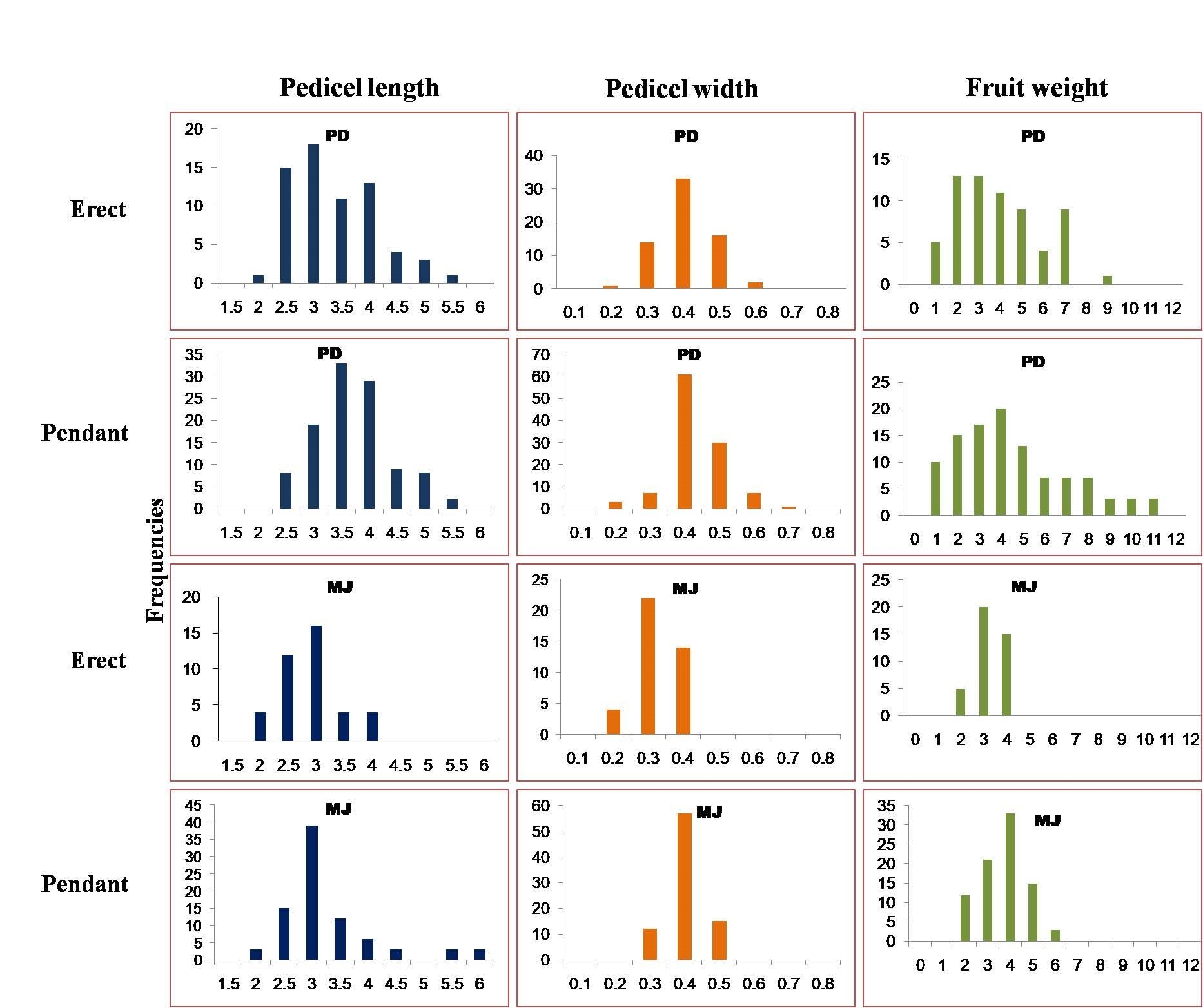

Supplement: Supplementary Figure 2 — Frequency distribution of traits related to fruit orientation. Pedicel length, pedicel thickness (width), and fruit weight of the PD and MJ F2 populations associated with the erect and pendent fruit orientations. [file Image_2.JPEG]

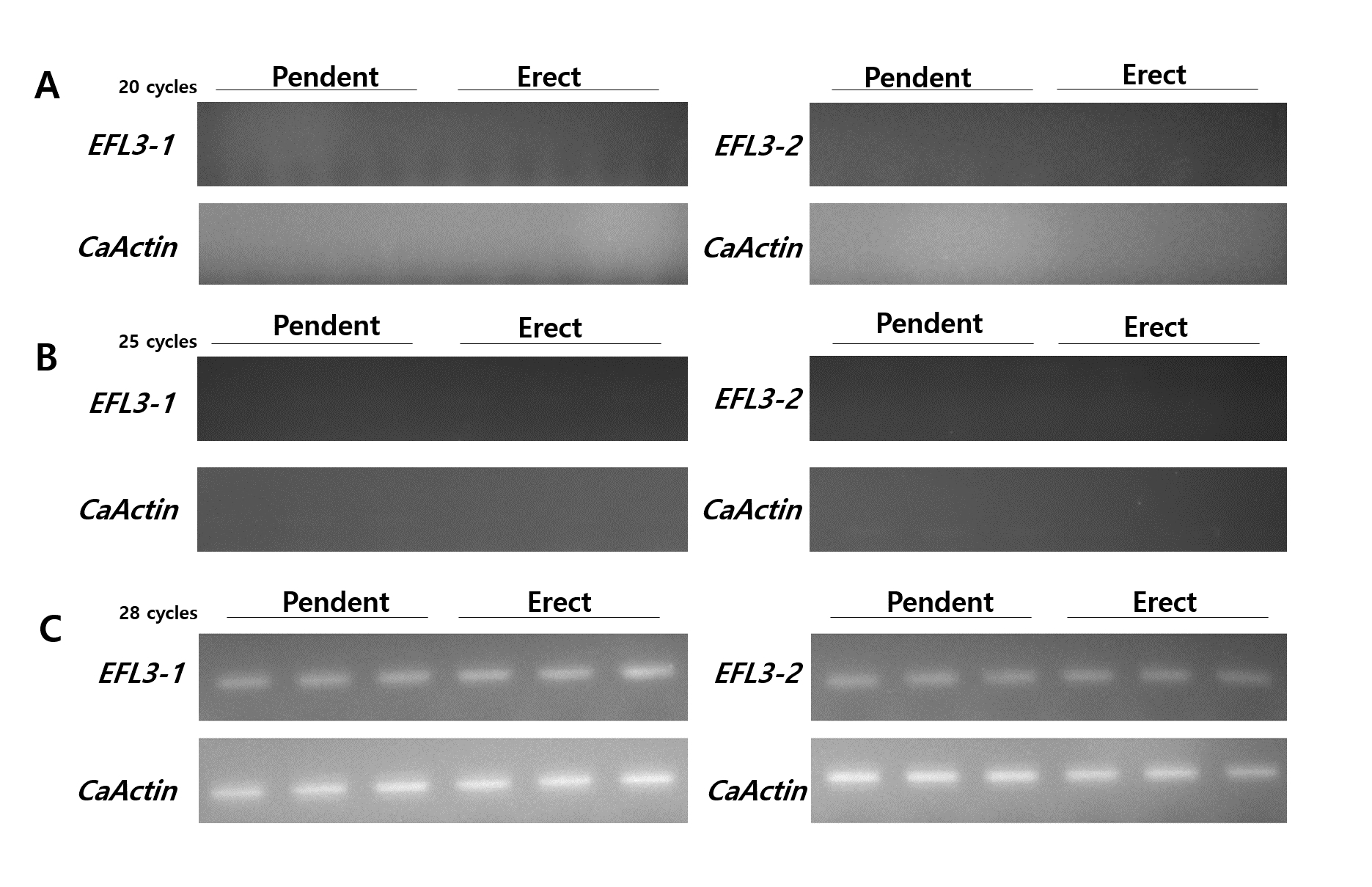

Supplement: Supplementary Figure 3 — Expression analysis of EFL3-1 and EFL3-2 in UB7 (erect) and GB57 (pendent). (A,B) In the pedicel of ‘UB7 and GB57’, EFL3-1, EFL3-2, and CaActin were not expressed. (C) EFL3-1 and EFL3-2 expression levels were identified. [file Image_3.JPEG]

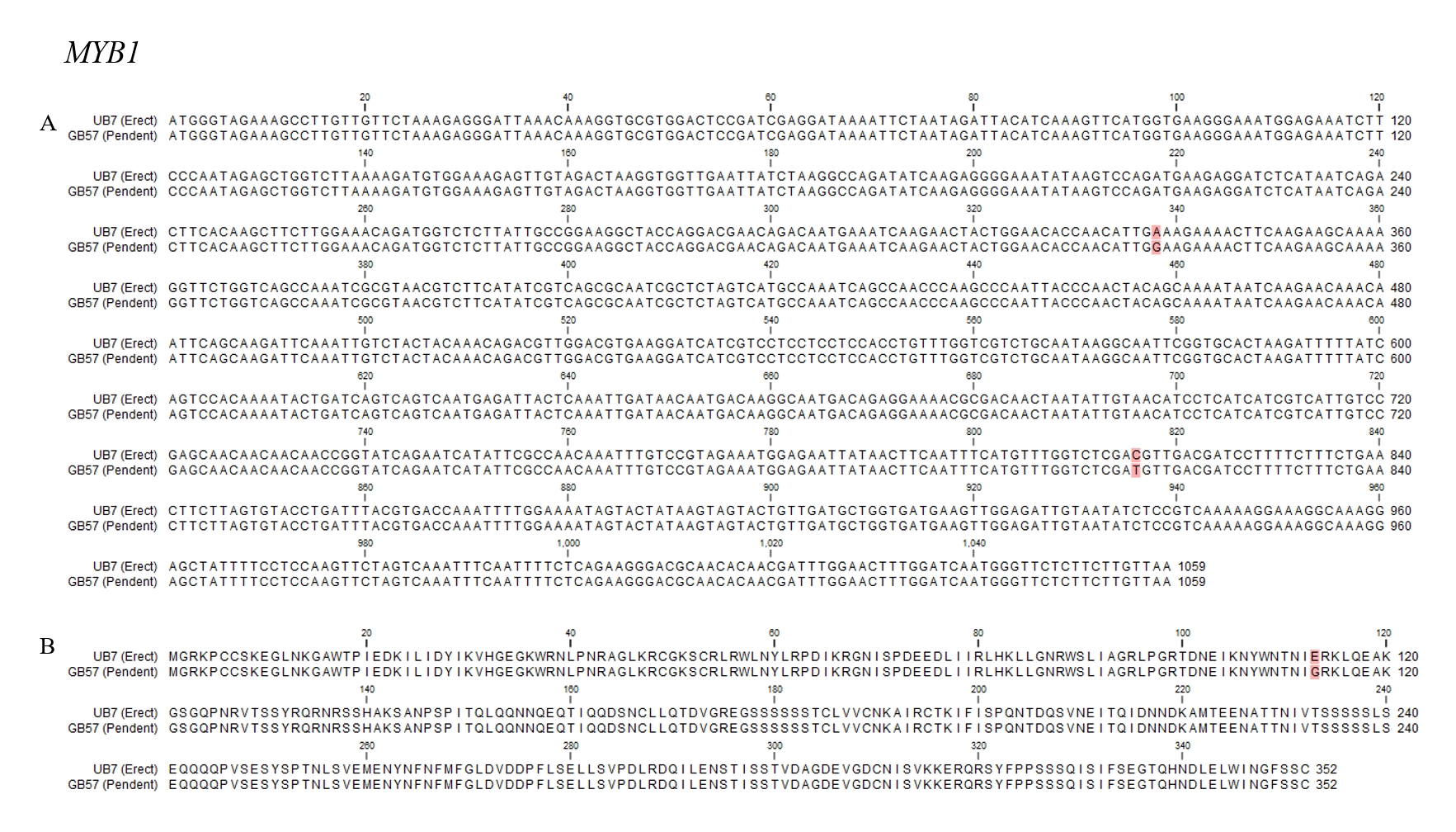

Supplement: Supplementary Figure 4 — Sequence variation analysis of MYB1. (A) Nucleotide sequence alignment. (B) Amino acid sequence alignment. [file Image_4.JPEG]

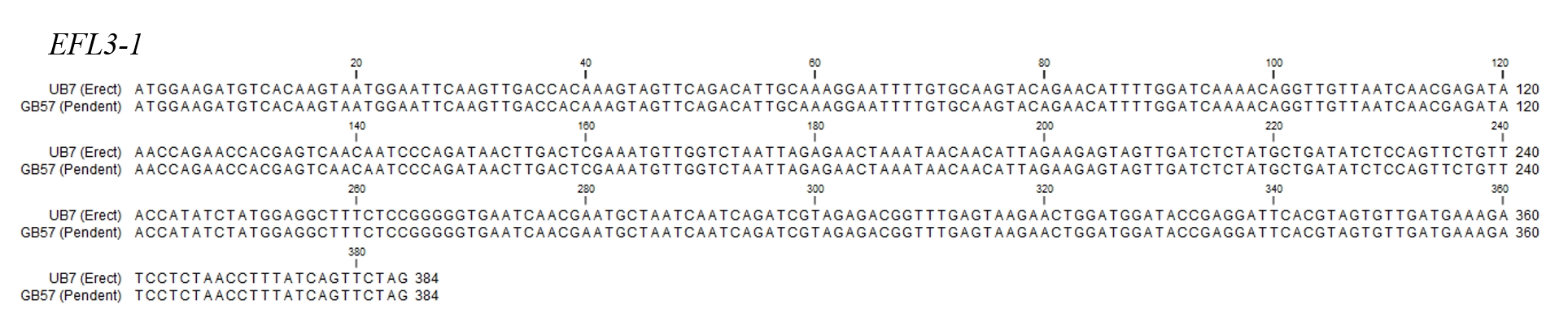

Supplement: Supplementary Figure 5 — Alignment of all EFL3-1 exon sequences. [file Image_5.JPEG]

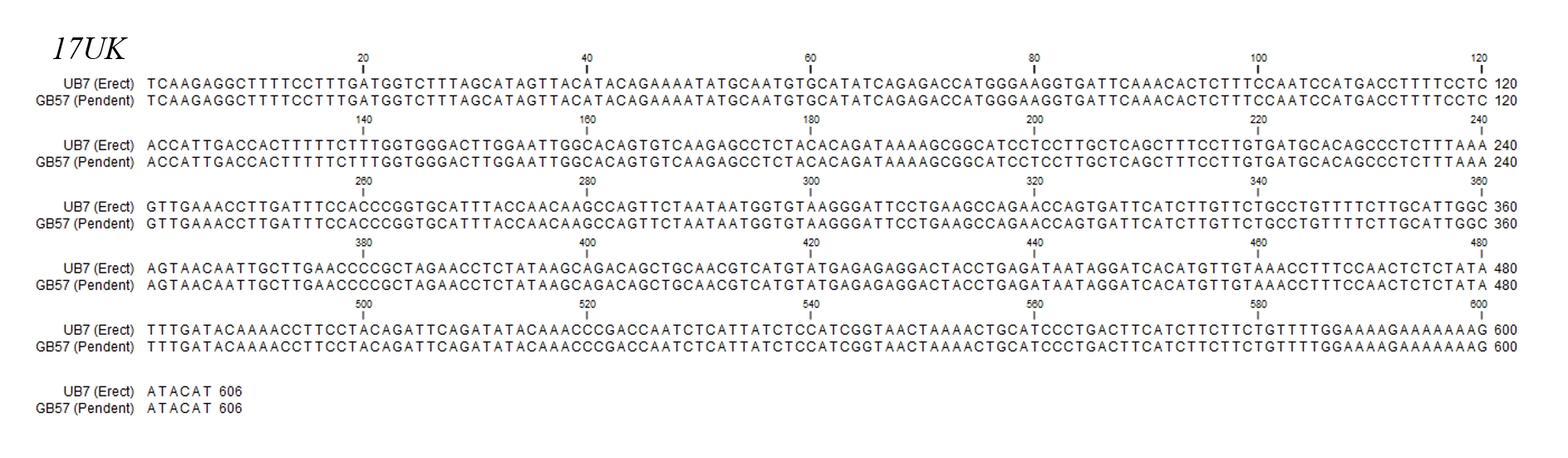

Supplement: Supplementary Figure 6 — Alignment of all 17UK exon sequences. [file Image_6.JPEG]

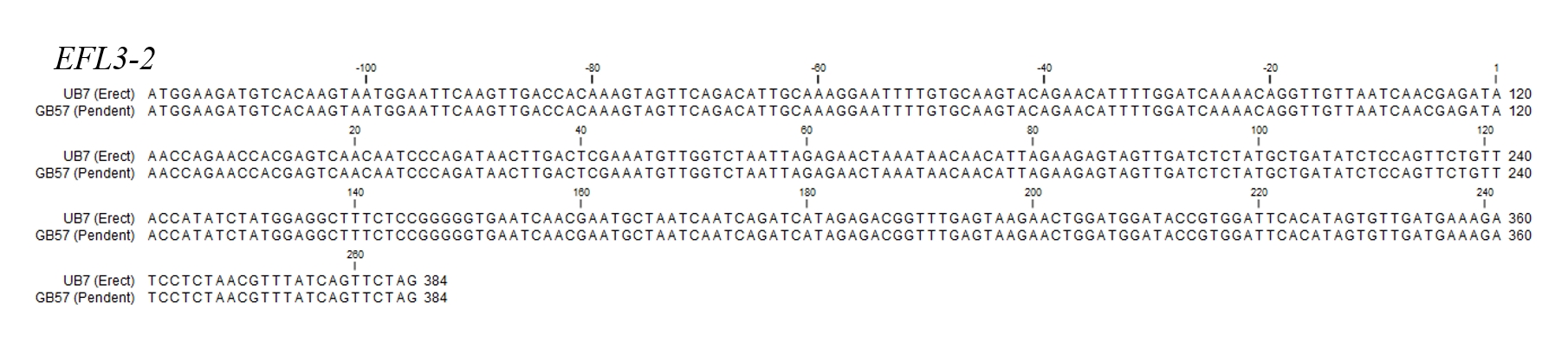

Supplement: Supplementary Figure 7 — Alignment of all EFL3-2 exon sequences. [file Image_7.JPEG]

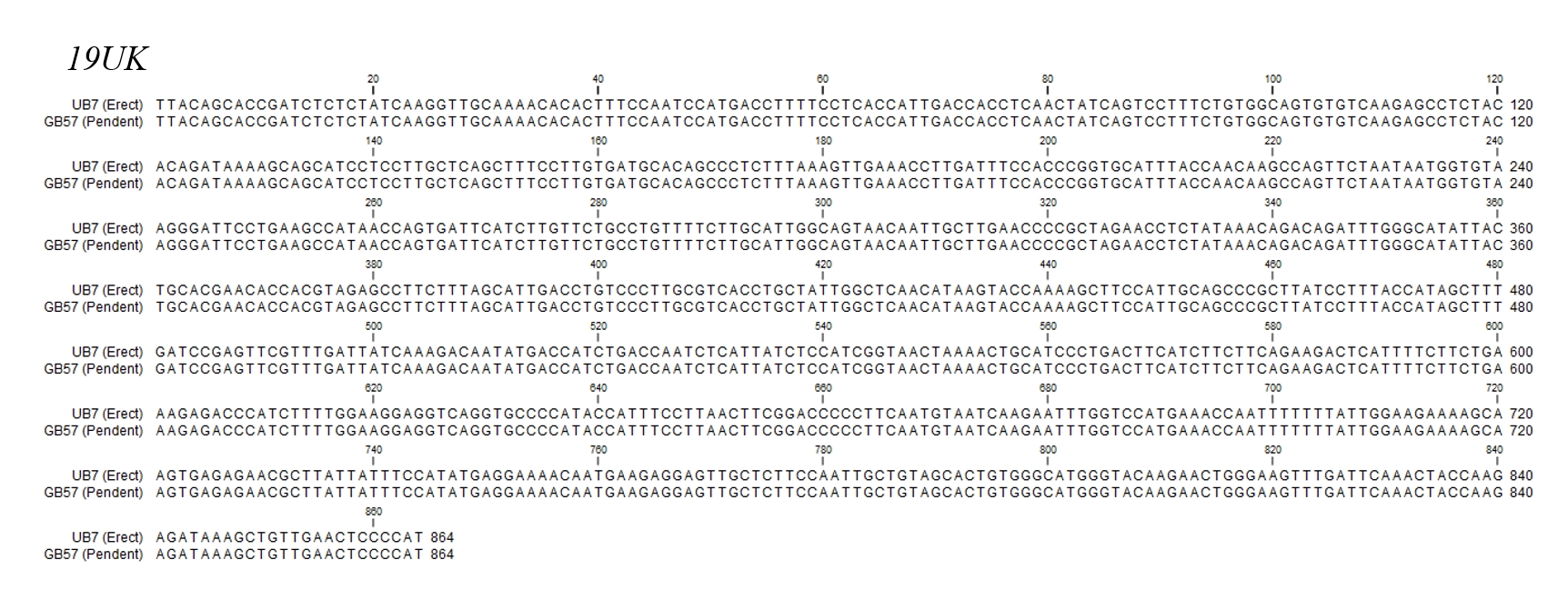

Supplement: Supplementary Figure 8 — Alignment of all 19UK exon sequences. [file Image_8.JPEG]

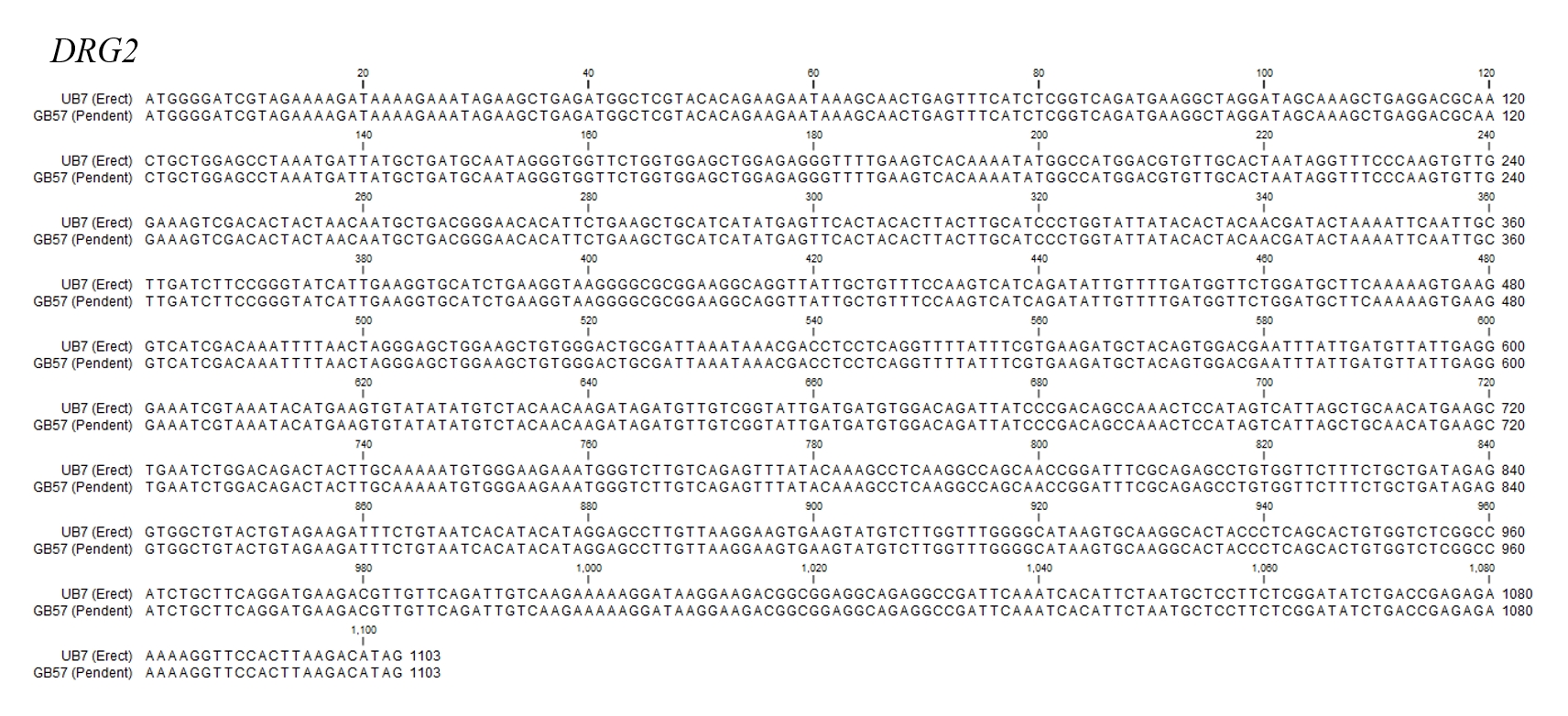

Supplement: Supplementary Figure 9 — Alignment of all DRG2 exon sequences. [file Image_9.JPEG]
