# Supplementary material for: Fine Mapping and Candidate Gene Identification for the CapUp Locus Controlling Fruit Orientation in Pepper (Capsicum spp.)
Source: Front Plant Sci. 2021 Jun 28;12:675474. doi: 10.3389/fpls.2021.675474 (PMC8273576; doi:10.3389/fpls.2021.675474)
Supplement: Supplementary Table 1 — Pearson correlation matrix of fruit orientation–related traits in the PD lines. [file Data_Sheet_1.docx]

**Supplementary tables**

**Table S1.** Pearson correlation matrix of fruit orientation–related traits in the PD lines.

| **Variables** | **Pedicel**  **width (cm)** | **Fruit**  **weight (g)** | **Pendent fruit orientation** |  |
| --- | --- | --- | --- | --- |
| Pedicel length (cm) | –0.021 | 0.164* | 0.222* |  |
| Pedicel width (cm) |  | 0.137 | –0.069 |  |
| Fruit weight (g) |  |  | 0.135 |  |
| * Significant positive relationship at *P* < 0.05 | | | | |

**Table S2.** Primers used in this study.

| **Primer name** | **Sequence** | **Sequence position**  **on chromosome 12** | **Amplicon**  **size (bp)** |
| --- | --- | --- | --- |
|  |  | **Dempsey v. 1.0** |  |
| DLMT218_191 | F: GGATTCTCGGGGTATTACAGG | 218193136 | 192 |
|  | R: TGTACCCTCCCCAGGTAGTAAG |  |  |
| UP199_462 | F: CTTTGGACCCCATTCTGATG | 219677187 | 189 |
|  | R: TTTCTTAAACCCGGATAAGCTG |  |  |
| UP199_942 | F: CGACGACCATCGTGTCTAAG | 220233699 | 230 |
|  | R: CCTCATTCCCTGCATTTTGT |  |  |
| UPKI541 | F: GCATGTCCCAACACTTTGTG | 220775872 | 210 |
|  | R: TACCAAATGCACCACACCTAAG |  |  |
| Kidus13-1 | F: ATGGCTATATGGGGATCAATCT | 221725211 | 250 |
|  | R: AGTGCTTTTGCAAGTATGAAGT |  |  |
| RSM_+28KB | F: GTCATAGAAAAATGGCTTGC | 221727694 | 249 |
|  | R:TTGAGAAGAGGTGTGATGAGATAAG |  |  |
