# Supplementary material for: Fine Mapping and Candidate Gene Identification for the CapUp Locus Controlling Fruit Orientation in Pepper (Capsicum spp.)
Source: Front Plant Sci. 2021 Jun 28;12:675474. doi: 10.3389/fpls.2021.675474 (PMC8273576; doi:10.3389/fpls.2021.675474)
Supplement: Supplementary file 18 [file Data_Sheet_8.PDF]

```
source("http://www.bioconductor.org/biocLite.R")
biocLite("multtest")
install.packages("gplots")
install.packages("LDheatmap")
install.packages("genetics")
install.packages("EMMREML")
install.packages("scatterplot3d")    #The downloaded link at:    http://cran.r-project.org/package=scatterplot3d
source("https://bioconductor.org/biocLite.R")
biocLite("qvalue")

library(multtest)
library(gplots)
library(LDheatmap)
library(genetics)
library(EMMREML)
library(compiler)
library("scatterplot3d")
source("http://zzlab.net/GAPIT/gapit_functions.txt")
source("http://zzlab.net/GAPIT/emma.txt")
setwd("C:\\myGAPIT")
```

```
#Step 1: Set data directory and import files
myCV <- read.table("Qmatrix.txt", head = TRUE)
myY    <- read.table("Phenotype.txt", head = TRUE)
myG <- read.table("Genotype.txt" , head = FALSE)

#Step 2: Run GAPIT
myGAPIT_SUPER <- GAPIT( Y=myY,
                        G=myG,
                        #KI=myKI,
                        CV=myCV,
                        #PCA.total=3,
                        sangwich.top="MLM", #options are GLM,MLM,CMLM, FaST and SUPER
                        sangwich.bottom="SUPER", #options are GLM,MLM,CMLM, FaST and SUPER
                        LD=0.1, )

library(car)
library(qqman)
GWAS.Manhattan<-data.frame(SNP=GWAS.Results$SNP,    CHR=GWAS.Results$Chromosome,    BP=GWAS.Results$Position, P=GWAS.Results$P.value, zscore=GWAS.Results$FDR)
```

```
manhattan(GWAS.Manhattan, p = "zscore", logp = FALSE, ylab = "-log10(FDR)", cex.axis = 0.7, genomewideline = 1.3, col = c("blue4", "green3", "red"), chrlabs = c("1A", "1B", "1D", "2A", "2B", "2D", "3A", "3B", "3D", "4A", "4B", "4D", "5A", "5B", "5D", "6A", "6B", "6D", "7A", "7B", "7D"),
          suggestiveline = 1, main = "TITLE")
```
